# The Mechanochemical Synthesis and Activation of Carbon‐Rich *π*‐Conjugated Materials

**DOI:** 10.1002/advs.202105497

**Published:** 2022-01-20

**Authors:** Mingjun Xuan, Christian Schumacher, Carsten Bolm, Robert Göstl, Andreas Herrmann

**Affiliations:** ^1^ DWI – Leibniz Institute for Interactive Materials Forckenbeckstr. 50 Aachen 52056 Germany; ^2^ Institute of Technical and Macromolecular Chemistry RWTH Aachen University Worringerweg 1 Aachen 52074 Germany; ^3^ Institute of Organic Chemistry RWTH Aachen University Landoltweg 1 Aachen 52074 Germany

**Keywords:** carbon‐rich materials, mechanosynthesis, polymer mechanochemistry, trituration, *π*‐conjugation

## Abstract

Mechanochemistry uses mechanical force to break, form, and manipulate chemical bonds to achieve functional transformations and syntheses. Over the last years, many innovative applications of mechanochemistry have been developed. Specifically for the synthesis and activation of carbon‐rich *π*‐conjugated materials, mechanochemistry offers reaction pathways that either are inaccessible with other stimuli, such as light and heat, or improve reaction yields, energy consumption, and substrate scope. Therefore, this review summarizes the recent advances in this research field combining the viewpoints of polymer and trituration mechanochemistry. The highlighted mechanochemical transformations include *π*‐conjugated materials as optical force probes, the force‐induced release of small dye molecules, and the mechanochemical synthesis of polyacetylene, carbon allotropes, and other *π*‐conjugated materials.

## Graphical abstract

Mechanochemistry allows the manipulation of chemical bonds by force as unique stimulus to impart functionality and bottom‐up bond formation. This allows the synthesis and activation of *π*‐conjugated carbon‐rich materials that would otherwise not be accessible. The authors highlight notable examples from the realms of polymer and trituration mechanochemistry.

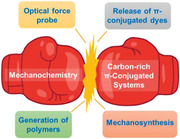

## 1 Introduction

Using mechanical force to activate chemical bond transformations enables reactions that cannot proceed using other established physical stimuli, such as light and heat.^[^ ^1^ ^]^ Being a fast‐growing and multipurpose technology,^[^ ^1^ ^]^ mechanochemistry was selected as one out of ten world‐changing innovations.^[^ ^2^ ^]^ Therefore, using mechanochemistry to synthesize and activate carbon‐rich nanomaterials was anticipated and one of the earliest examples can be traced back to the late 1990s.^[^ ^3^ ^]^ According to Suslick, mechanochemistry can be divided into four areas that are distinct but related: tribochemistry, trituration, polymer mechanochemistry, and sonochemistry.^[^ ^4^ ^]^ While aspects of these subfields overlap, the techniques used for defined synthetic purposes are mostly found in polymer as well as trituration mechanochemistry.

Polymer mechanochemistry relies on the extension of macromolecular termini that are substituted to individually defined positions of the reacting molecular motif, the mechanophore.^[^ ^5^ ^]^ In this instance and unlike heat, force acts as a vector quantity, that is, it is intrinsically directional regarding bond orientation. This allows precise localization of the force and hence introduces a sense of directionality by “force‐coupling” certain bonds within the mechanophore.^[^ ^6^ ^]^ Consequentially, most reactions in polymer mechanochemistry known today rely on the extension‐induced selective bond scission as the initial step of the mechanochemical reaction. However, polymer mechanochemistry is more than a collection of destructive processes as the downstream formation of new bonds can be achieved, for example, by the force‐activation of latent catalysts,^[^ ^7^ ^]^ self‐healing functions,^[^ ^8^ ^]^ complex reaction cascades,^[^ ^9^ ^]^ or amplified stimulus–response feedback loops.^[^ ^10^ ^]^

An alternative approach for the bottom‐up synthesis and functionalization of molecules and materials is the use of trituration mechanochemistry,^[^ ^1^ ^]^ which is defined as the realization of chemical reactions by direct absorption of mechanical energy exerted by grinding and milling.^[^ ^11^ ^]^ In contrast to polymer mechanochemistry, trituration mechanochemistry relies on the compression and shearing of molecules and hence is mostly associated to bond formation reactions. Compared to solution‐based protocols, this approach offers distinct advantages, such as reduced reaction time and solvent usage,^[^ ^12^ ^]^ cleaner and greener reactions,^[^ ^12^ ^]^ access to products that are inaccessible by other synthetic protocols,^[^ ^13^ ^]^ or reactions of insoluble materials.^[^ ^14^ ^]^ Trituration mechanochemical reactions can be performed by kneading, stretching, shearing, pressing, or grinding substances.^[^ ^11^, ^12^, ^13^ ^]^ Often, it involves the use of ball mills allowing the accomplishment of a mechanochemical reaction under controlled and reproducible conditions compared to the classical grinding using a mortar and pestle.^[^ ^14^ ^]^ The toolbox of trituration mechanochemistry expands constantly as prospects for cooling,^[^ ^15^ ^]^ heating,^[^ ^16^ ^]^ in situ spectroscopy,^[^ ^17^ ^]^ liquid assisted grinding (LAG),^[^ ^18^ ^]^ or the implementation of gaseous reagents are developed.^[^ ^19^ ^]^

This review focuses on mechanochemical methods and reactions to activate and synthesize carbon‐rich and *π*‐conjugated materials and highlights illustrative examples thereof. We will guide the reader through the polymer mechanochemical activation of acene‐ and rylene‐based optical force probes (OFPs) and the release of *π*‐conjugated dyes. Hereafter, we turn to discuss the polymer mechanochemical synthesis of polyacetylene derivatives and the trituration mechanosynthesis of hexabenzocorones, related carbon materials, and allotropes. We then briefly summarize the pertinent future challenges in this field. The plethora of both polymer and trituration mechanochemical syntheses and activation reactions of carbon‐rich materials is an extensive and significantly growing field. Hence, this review will focus on selected and transformative illustrations from recent years and prestigious examples will be reviewed regarding their potential applications.

## 2 Acene‐ and Rylene‐Based Optical Force Probes for Polymer Mechanochemistry

### 2.1 The Optical Force Probe Principle

Incorporating OFPs into polymer architectures allows the detection and visualization of force‐induced events and their interrelation with material mechanics. OFPs undergo molecular transformations leading to molecular optical changes upon the mechanical deformation of the bulk polymer material they are incorporated within, including compression, extension, twisting, and folding. Therefore, OFPs are rendered useful molecules to detect the effects of mechanical force in tough high‐performance polymers as well as in soft biomimetic systems.^[^ ^20^ ^]^

OFPs detect the force‐induced alterations of chemical bonds, structures, and spatial conformations by changes of absorption, fluorescence, or chemiluminescence. OFPs allow a real‐time in situ or post mortem monitoring to investigate mechanical transformations. They can detect breaking covalent bonds (nanonewton range) as well as the dissociation of non‐covalent interactions (piconewton range), depending on the employed molecular motif and the polymer architecture they are anchored within.^[^ ^6^ ^]^

**Figure 1:**
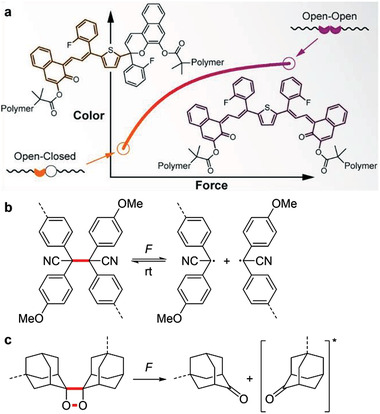
Different OFP types. a) Mechanochromogenic OFPs: The bis‐naphthopyran polymer exhibits a chromogenic response upon mechanical activation. Reproduced with permission.^[^ ^22^ ^]^ Copyright 2019, American Chemical Society. b) Mechanofluorescent OFPs: mechanically triggered transformation between colorless tetraarylsuccinonitrile and pink carbon‐centered radicals that emit yellow light under UV irradiation.^[^ ^25^ ^]^ c) Mechanoluminescent OFPs: schematic illustration of mechanically induced luminescence based on bis(adamantyl)‐1,2‐dioxetane.^[^ ^26^ ^]^ Mechanochemically active bonds are bold red and polymer attachment points are dashed lines—here and throughout the review.

Mechanochromogenic (i.e., changes in absorption) detection is popular to investigate mechanical transformations of polymers through visualization on the whole sample level. Spiropyran‐based OFPs were the first mechanochromogenic systems reported by Sottos, Moore, White, and coworkers, where local color and fluorescence displayed changes in stretched elastomeric and glassy polymers.^[^ ^21^ ^]^ This work influenced later OFP designs and applications and hence various mechanochromogenic OFPs have been developed, such as naphthopyran, oxazine, or persistent radicals (e.g., diarylbibenzofuranone). These OFPs are chemically modified and covalently anchored within the polymer architecture. For example, a bis‐naphthopyran OFP‐centered polymer can be activated by sonication and exhibits force‐induced color changes in the visible light range (**Figure** **1**a).^[^ ^22^ ^]^ Alongside, the naphthopyran OFP can also covalently crosslink polydimethylsiloxane (PDMS) which enables its reversible multicolor response to force.^[^ ^23^ ^]^ In addition to force detection, multicolor chromism and the discrimination of several types of mechanical stimuli can be achieved using OFPs. Therefore, two polymers were blended by Otsuka and coworkers comprising different radical‐type OFPs, tetraarylsuccinonitrile and diarylbibenzothiophenonyl, which afforded the corresponding radicals, diarylacetonitrile and arylbenzothiophenonyl, by mechanically cleaving the central C—C bond of the OFPs and a color change to discriminate stretching and grinding.^[^ ^24^ ^]^

Although mechanochromogenic OFPs successfully detect force distributions, they are less sensitive regarding spatiotemporal resolution compared to emission changes. For example, mechanofluorogenic OFPs offer ultrasensitive detection and visibility by transforming from a silent into an active state or from low to high fluorescence. This is not only suitable to detect bond cleavage events but also to monitor non‐covalent interactions, including hydrogen bonds, *π*‐attractive forces, and metal‐ligand interactions. A notable work by Sottos, Moore, White, and coworkers employs a force‐sensitive spiropyran OFP to crosslink poly(methyl methacrylate) (PMMA) networks. This system makes use of both absorption and fluorescence changes when the electrocyclic ring‐opening reaction is activated by shear force.^[^ ^27^ ^]^ Moreover, tetraarylsuccinonitrile derivatives as covalent fluorogenic OFPs were incorporated by Otsuka and coworkers into polymer/silsesquioxane composites, where stable pink radicals and yellow‐light emission were observed in a sol‐gel system under UV illumination as central C—C bonds were mechanically cleaved (Figure 1b).^[^ ^25^ ^]^ In addition, mechanofluorogenic OFPs can detect force ratiometrically. Salaita and coworkers reported a fluorophore‐quencher system formed by folding a DNA hairpin, which is optically sensitive to a tunable force response. When a piconewton‐force is applied to unfold the hairpin, a 20–30‐fold fluorescence increase could be achieved.^[^ ^28^ ^]^ A comparable design was shown by Walther and coworkers in a 3D DNA gel with a Förster resonance energy transfer (FRET)‐based fluorophore‐quencher system relying on the force‐induced dissociation of DNA base pairs to separate the dyes.^[^ ^29^ ^]^

Different from the above two types of OFPs, mechanoluminescent OFPs can be considered the most sensitive regarding spatiotemporal resolution. The only motif known today, bis(adamantyl)‐1,2‐dioxetane, emits bright‐blue luminescence upon activation by force (Figure 1c).^[^ ^26^, ^30^ ^]^

### 2.2 Acene‐Based Optical Force Probes

#### 2.2.1 [4+4] Cycloadducts (Anthracene Dimers)

Acenes are polycyclic aromatic hydrocarbons (PAHs) with linearly fused benzene rings. The outstanding attention acenes receive is rooted in their unique optoelectronic properties stemming from their intrinsic *π*‐bond topology, which results in applications in electronics, optics, and semiconductors.^[^ ^31^ ^]^ Anthracene is one of the smaller acenes which contains three linear aromatic benzene units. Anthracenes have a comparably low HOMO–LUMO gap due to their planar and extended aromatic *π*‐system consisting of 14 delocalized electrons. Thus, the excited state can be populated easily upon illumination with UV light.^[^ ^32^ ^]^ Most anthracenes and their derivatives are suitable fluorophores because of their high fluorescence quantum yields and nanosecond fluorescence lifetimes.

**Figure 2:**
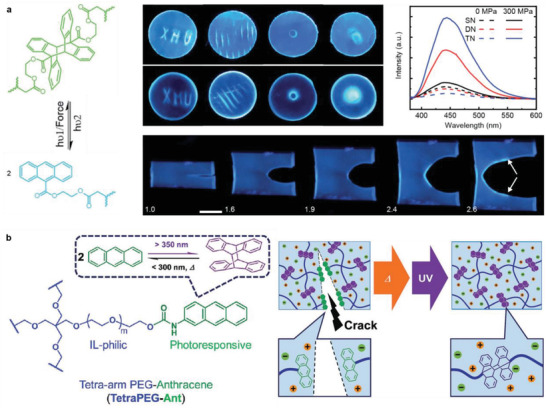
Dimeric anthracene derivatives as mechanofluorogenic OFPs for crack detection by cycloelimination. a) Anthracene dimer OFP crosslinks multi‐network elastomers to detect compression and crack propagation. Adapted with permission.^[^ ^34^ ^]^ Copyright 2019, Royal Society of Chemistry. b) Four‐armed PEGylated anthracene ionogel with automatic crack sensing and healing properties. Adapted with permission.^[^ ^35^ ^]^ Copyright 2018, Royal Society of Chemistry.

Two anthracene molecules can fuse to an eight‐membered ring, dimeric anthracene, through a photochemical reaction, which is the earliest reported example of a [4+4] photocycloaddition. Different from pristine anthracene, dimeric anthracene is not fluorescent. Therefore, the transformation of an anthracene dimer into anthracene endows it with OFP character for the detection of covalent mechanical bond scission, as occurs during cracking, stretching, and compression. An early study by Chung and coworkers relied on dimerized 9‐anthraldehyde and 9‐anthracenecarboxylic acid. The two resulting OFPs covalently crosslink poly(vinyl alcohol) (PVA) gels and exhibit strong fluorescence when cracking occurs.^[^ ^33^ ^]^ In addition, anthracene dimer OFPs have been incorporated into crosslinked stiff double‐ and triple‐network elastomers by Weng, Creton, Boulatov, and coworkers.^[^ ^34^ ^]^ The elastomers are mechanochromic with both persistent and reversible fluorescence by regenerating anthracene dimers under UV light after bond scission (**Figure** **2**a). Additionally, four‐armed poly(ethylene glycol) (PEG)‐substituted anthracene was synthesized to form an ionogel by dimerization.^[^ ^35^ ^]^ This system could not only emit fluorescence upon cracking, but also exhibited a healing function to recover cracked regions after heating and UV irradiation (Figure 2b).

#### 2.2.2 [4+2] Cycloadducts (Diels–Alder Adducts)

In addition to dimerization, anthracene can form [4+2] cycloadducts with suitable dienophiles, such as maleimide derivatives. Similar to [4+4] cycloadducts, these inhibit fluorescence by disconnecting the *π*‐systems of the diene and are hence useful as OFPs. When anthracene‐maleimide adducts are cleaved by force, the anthracene returns to its fluorescent state.

**Scheme 1:**
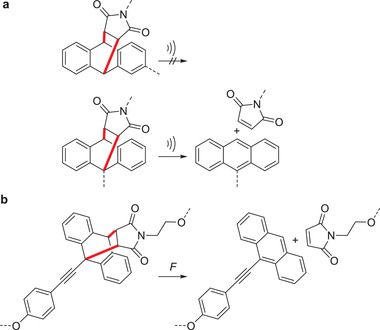
OFPs based on anthracene‐maleimide cycloadducts to detect force‐induced bond scission events. a) The cycloelimination proceeds when the polymer is substituted to the 9‐position of the anthracene but not on the 2‐position.^[^ ^36^ ^]^ b) Adducts of *π*‐extended anthracenes and maleimide serve as sensitive, color‐tunable OFPs.^[^ ^39^ ^]^

[4+2] cycloadducts of anthracene and maleimide as OFPs report bond scission events under force. However, this only works when the attachment point of the polymer chain to the OFP is chosen correctly. For example, the mechanical reactivity of such OFPs was investigated both by computation and measurements and it was shown that polymer substitution in the 9‐position of the anthracene led to successful force‐induced activation of the motif, while substitution in 2‐position did not (**Scheme** **1**a).^[^ ^36^ ^]^ However, the dependence of mechanochemical reactivity on the polymer structure is not straightforward. A study by Boydston and coworkers concluded that three‐armed star chains and linear polymers show comparable scission rate constants.^[^ ^37^ ^]^ Furthermore, anthracene‐maleimide Diels–Alder adduct OFPs have been used by Moore and coworkers to detect force‐induced interfacial bond scission of nanoparticles decorated with polymer chains.^[^ ^38^ ^]^

Diels–Alder adducts of *π*‐extended anthracenes and maleimide recently gained importance as useful OFPs (Scheme 1b).^[^ ^39^ ^]^ Notably, adding the triplet sensitizer platinum octaethylporphyrin (PtOEP) to these OFPs allows the detection of bond scission by triplet‐triplet annihilation photon upconversion of green to blue light both solution and the solid state.^[^ ^40^ ^]^ The *π*‐extension in 9‐position of the anthracene allows for easy spectral tuning and hence dual fluorescent OFPs could be realized, which aided in the development of quantitative bond scission detection protocols.^[^ ^41^ ^]^ These Diels–Alder adduct OFPs proved valuable for various applications, including 3D fluorescent damage mapping with high spatial resolution, precise quantification of bond scission events, and qualitative OFP‐assisted fractography.^[^ ^42^ ^]^

### 2.3 Rylene‐Based Optical Force Probes

Rylene and rylene derivatives are PAHs, which are based on naphthalene units linked in the *peri*‐position.^[^ ^43^ ^]^ Rylenes are popular emitters since they exhibit high molar absorptivities, high fluorescence quantum yields, and remarkable chemical stability. Their large *π*‐conjugated structures offer a versatile synthetic basis for their application as electron acceptors, color‐turning dyes, fluorescent probes for cellular imaging and protein tagging, and in organic field‐effect transistors as well as photovoltaics.

Due to their excellent optical properties, considerable attention is devoted to utilizing rylenes as OFPs. Although mechanofluorochromic rylene‐doped polymers are among the first studied examples of reported mechanochromism, their use as force reporters anchored within the polymer architecture (i.e., OFPs) is still in its infancy. Rylene OFPs are usually activated by altering non‐covalent interactions, which distinguish them from OFPs based on covalent bond scission. Their force‐modulated optical response relies on the alteration of *π*–*π* interactions between the conjugated planar structures or hydrogen bonding interactions among the functional peripheral moieties. Early studies by Pucci, Ruggeri, and coworkers report that perylene derivatives were blended within linear low‐density polyethylene. With the application of mechanical force, *π*–*π* intermolecular interactions between their supramolecular aggregates were dissolved resulting in the alteration of their emission from red to green.^[^ ^44^ ^]^ Yet such blends were not molecularly defined and in addition to occurring multimolecular aggregates, the force‐induced optical alterations were simultaneously the result of intra‐ and/or intermolecular rearrangements. Thus the molecular alterations were not defined enough to render them true OFPs.

**Figure 3:**
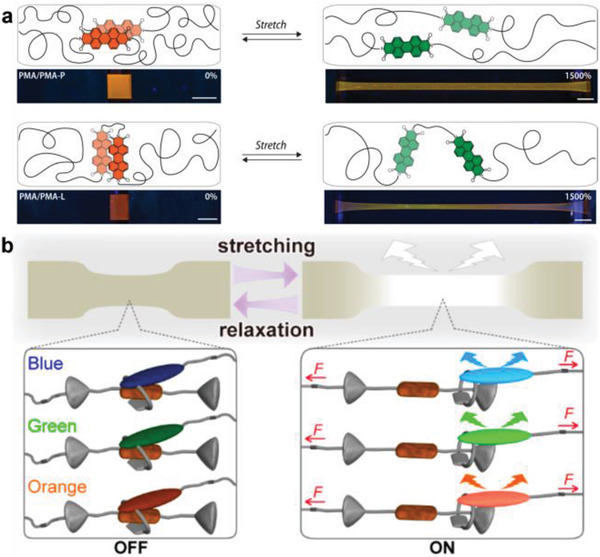
Rylene‐based OFPs. a) The activation of excimer‐forming perylene diimide loops. Reproduced with permission.^[^ ^45b^ ^]^ Copyright 2021, Wiley‐VCH. b) An interlocked rotaxane OFP composed of a fluorescent macrocycle and a linear dumbbell‐shaped naphthalene diimide quencher for force visualization. The rotaxanes contain cycles with attached fluorophores (blue, green, orange) and a matching quencher (brown) in the axle of a dumbbell‐shaped molecule with two stoppers (gray). Reproduced with permission.^[^ ^47^ ^]^ Copyright 2019, American Chemical Society.

However, rylene mechanofluorophores can also be used as OFPs as their activation by charge‐transfer interactions recently showed. Therefore, fluorescent pyrene and naphthalene diimide were connected by a flexible spacer to form a tandem structure, which was incorporated into the middle of a poly(*ε*‐caprolactone) chain. The fluorescence of pyrene is temporally quenched due to the formation of an intramolecular charge‐transfer complex. The application of force separates both fluorophores effectively abandoning the charge‐transfer state.^[^ ^45a^ ^]^ Building on this concept, Weder, Schrettl, and coworkers connected two molecules of the excimer‐forming dye perylene diimide by a short poly(methyl acrylate) chain thus rendering this system an OFP.^[^ ^45b^ ^]^ In the equilibrium state this led to the formation of folded loop structures in the polymer matrix, where the emission was dominated by excimers (**Figure** **3**a). Upon stretching of the OFPs, this system undergoes a visually discernible fluorescence color change as the loops unfold.

Besides covalent connection, rylene‐based OFPs were constructed by using the mechanical bond. Therefore, an interlocked rotaxane containing a fluorescent macrocycle and a linear dumbbell‐shaped molecule as a quencher were prepared by Weder, Sagara, and coworkers. By incorporation of this structure into polyurethane (PU) elastomer and the subsequent application of force, this OFP modulated its emission signal due to the mechanically induced spatial separation of fluorophore and quencher within the mechanical bond (interlocking) of the rotaxane.^[^ ^46^ ^]^ This system was subsequently enhanced using three different types of *π*‐extended fluorescent macrocycles (Figure 3b), including pyrene, anthracene, or 4‐(dicyanomethylene)‐2‐methyl‐6‐(4‐dimethylaminostyryl)‐4*H*‐pyran luminophores, respectively, to match the quencher 1,4,5,8‐naphthalenetetracarboxylic diimide, to achieve a broad color variation for the detection of force‐induced events.^[^ ^47^ ^]^ This class of interlocked OFPs is quite flexible and was further diversified by the alteration of the emitting fluorescent macrocycle and the linear backbones of the quenchers.^[^ ^48^ ^]^ Through the activation of interlocked OFPs, specific, efficient, tunable, and reversible alterations of the optical signal under mechanical force can be obtained. However, this comes at the price of a rather complicated synthesis.

## 3 The Polymer Mechanochemical Release of *π*‐Conjugated Dyes

One of the masterpieces of polymer mechanochemistry is the design of processes where covalent chain scission (that normally yields two shorter polymeric fragments) leads to a release of a small molecule. Pioneered by Larsen and Boydston, who developed the “flex‐activation” approach,^[^ ^49^ ^]^ recent works contributed molecular designs that allow the fine‐tuning of cargo release kinetics. Naturally, such release processes can also be employed to release and activate emitting dye molecules.

The “flex‐activation” approach employs Diels–Alder adducts of acetylenedicarboxylates and furans. Larsen and Boydston applied force through the acetylenedicarboxylate backbone thus planarizing the mechanophore and releasing the furan derivative.^[^ ^49^, ^50^ ^]^ Yet this system showed limitations in terms of cargo scope, release efficiency, and was not proven to work in solution.

**Scheme 2:**
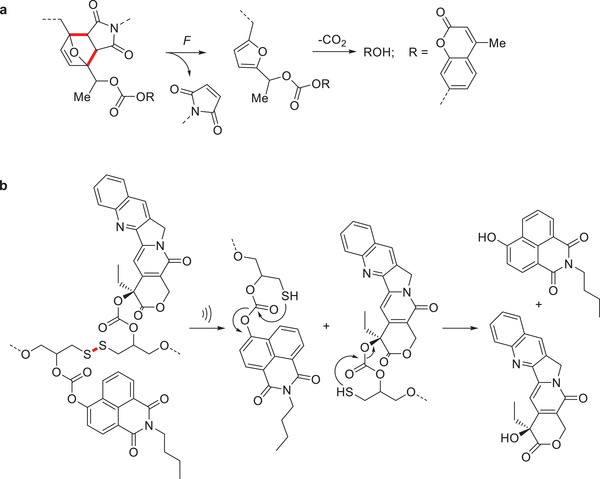
Release of *π*‐conjugated latent fluorophores from polymers based on mechanochemically activated cascade reactions. a) The furan‐maleimide DA adducts release a hydroxycoumarin derivative.^[^ ^51^ ^]^ b) Disulfide cleavage induced cargo release caused by mechanochemically induced 5‐*exo*‐*trig* cyclization.^[^ ^57^ ^]^

Recently, Robb and coworkers designed a mechanophore based on the Diels–Alder adduct of furan and maleimide such that the mechanochemical generation of the furyl moiety would spawn a metastable furfuryl carbonate. This would then quickly decompose and release CO_2_ and a hydroxyl‐functionalized molecule in polar protic media. This concept was successfully employed to release the latent *π*‐conjugated fluorophore hydroxycoumarin derivative as a probe molecule (**Scheme** **2**a).^[^ ^51^ ^]^ Further development allowed tuning both the release rates and cargo scope to include alkyl and aryl alcohols, amines, carboxylic acids, as well as sulfonic acids.^[^ ^52^ ^]^ When an electron donating phenoxy group was introduced in the 3‐position of the furan ring, the activation barrier for carbonate fragmentation of the furfuryl C—O bond was lowered resulting in a highly active substrate for cargo molecule release. The applicability of this was demonstrated with the swift release of aminocoumarin.^[^ ^53^ ^]^

Alongside, Herrmann, Göstl, and coworkers developed a release system based on the established disulfide mechanophore.^[^ ^54^ ^]^ Upon the application force, the disulfide bond is cleaved homolytically and in protic solvents two chain‐terminal thiols are generated. When oxanorbornadiene structures, comparable to those of Boydston and coworkers, react in a Michael‐addition with such a mechanochemically generated chain‐terminal thiol, the retro‐ Diels–Alder reaction is triggered and the release of a furan‐bearing compound proceeds. Besides pharmaceutically active compounds, this was demonstrated for the latent *π*‐conjugated fluorophore furan‐dansyl.^[^ ^55^ ^]^ A revised design was developed shortly afterwards where no intermolecular reactions are necessary. The disulfide mechanophore was positioned such that it bore carbonate linkers in *β*‐position. The subsequent mechanochemical formation of thiols then spawned an intramolecular 5‐*exo‐trig* cyclization releasing the cargo from the carbonate.^[^ ^56^ ^]^ This release system was then used to develop a theranostic drug delivery system where one dye molecule was released alongside one drug molecule. Therefore, umbelliferone and *N*‐butyl‐4‐hydroxy‐1,8‐naphthalimide could be released and activated as fluorophores in this process (Scheme 2b).^[^ ^57^ ^]^ Further expanding cargo scope, the possibility to release amino‐functionalized cargo from *β*‐carbamates was demonstrated as well by the release of fluorogenic amino‐naphthalimide derivatives.^[^ ^58^ ^]^ However, since the release from carbamates was found to be slow, the system developed by Robb and coworkers appears more suitable for amino‐functionalized cargo molecules.

Research into the polymer mechanochemical release of *π*‐conjugated dyes is still in the beginning stage and only few examples are documented within the literature. However, with view on the broad applicability of “turn‐on” fluorescent probes as sensors and biomarkers, the development of novel release mechanisms and cargo molecules is anticipated to be highly attractive.

## 4 The Polymer Mechanochemical Generation of Conjugated Polymers

**Scheme 3:**
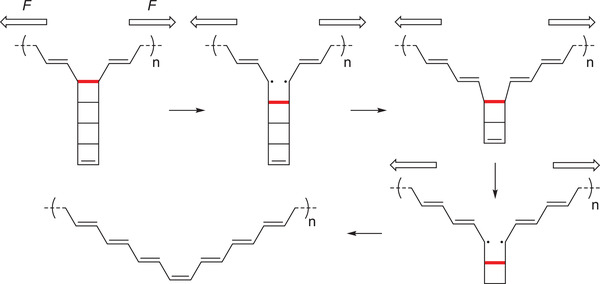
The mechanochemical transformation of polyladderene into polyacetylene.

**Figure 4:**
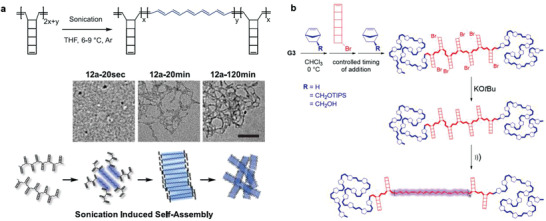
Generation of *π*‐conjugation based on the mechanochemistry of polymechanophores. a) Mechanochemical transformation from non‐conjugated polyladderenes to conjugated polyacetylene. Reproduced with permission.^[^ ^59^ ^]^ Copyright 2017, AAAS. b) Mechanochemical generation of ladderene‐norbornene block copolymers. Reproduced with permission.^[^ ^61^ ^]^ Copyright 2018, American Chemical Society.

One of the most notable achievements of the polymer mechanochemical generation of functional *π*‐systems is the synthetic access to polyacetylene structures by consecutive ring‐opening reactions of polyladderenes. Polyacetylene is the simplest conceivable *π*‐conjugated polymer and consists of a linear backbone with alternating single and double bonds. The conventional synthesis of polyacetylene is conducted by the ring‐opening metathesis polymerization (ROMP) of cyclooctatetraene, which represents a more straight‐forward approach compared to the direct polymerization of acetylene. Recently, Xia, Burns, Martínez, and coworkers devised a polymer mechanochemical strategy using polyladderene to activate consecutive cyclobutane cycloeliminations producing polyacetylene (**Scheme** **3**).^[^ ^59^ ^]^ Specifically, [5]‐ladderenes were polymerized to polyladderene by ROMP. The application of force sequentially unzipped the cyclobutyl rings of and caused bond conversion from strained *σ*‐ to *π*‐bonds, which successfully transformed non‐conjugated polyladderene to polyacetylene (**Figure** **4**a, top). In addition to the alteration of the electronic properties, the contour length of the main chain increased significantly upon unzipping caused by mechanochemical *Z*‐ to *E*‐isomerization of the central double bond. However, pure polyacetylene could not be obtained since the applied force was only above the activation threshold in the central region of the polyladderene chains resulting in incomplete unzipping in the chain periphery and the formation of ABA‐type triblock copolymers. As a result, self‐assembly of the resulting polymer chains led to the formation of nanostructures due to the interaction between polyacetylene and polyladderene blocks (Figure 4a, bottom).

In a related work, Frank investigated the mechanochemistry and photochemistry of [4]‐ladderene by computational simulations and further confirmed the anticipated mechanochemical transformation from polyladderene to polyacetylene. The threshold force to activate the transformation was determined to comparatively moderate 3 nn. In addition, it was found that the *E*‐ to *Z*‐isomerization strongly relied on steric factors.^[^ ^60^ ^]^ Circumventing the initial issues of the incomplete conversion and resulting self‐assembly, Xia and Burns synthesized ladderene‐norbornene block copolymers.^[^ ^60^ ^]^ In this system, active polyladderene blocks are incorporated within the center of two inactive polynorbornene chains to form triblock copolymers (Figure 4b). This terminal functionalization also enables the potential integration into diverse materials as crosslinkers.

Although polyladderene is a fascinating polymechanophore, its monomer precursor [5]‐chloro/bromoladderene suffers from complicated synthesis and low production yield. Xia and coworkers recently developed a new type of ladderene, namely benzoladderene, for scalable synthesis of mechanophore monomers with gram quantities. The ROMP of benzoladderenes also allows the formation of homopolymers and block copolymers with desired molar masses and the incorporation of simple functionalities. Upon force application by ultrasonication, non‐conjugated poly(benzoladderene) is ring‐opened to conjugated poly(*o*‐phenylene‐hexatrienylene).^[^ ^62^ ^]^

Despite the improvements achieved with benzoladderene, the synthesis of the monomer precursors still suffered from the problem of instable intermediates. To circumvent this, bicyclo[2.2.0]hex‐5‐ene‐2,3‐peri‐naphthalene (BCH‐Naph), was synthesized by using stable materials with a range of electron‐rich or ‐poor substituents.^[^ ^63^ ^]^ The ROMP of BCH‐Naph gives access to ultrahigh molar mass polymechanophores with controlled molar mass distributions and low dispersities.

To elucidate the underlying reaction mechanism of the unzipping reaction in more detail, Xia, Martínez, and coworkers connected [4]‐ladderane and [4]‐ladderene to several types of polymer backbones. They observed that the cascade mechanochemical activations did not yield any half‐unzipped intermediates and verified identical stereochemical distributions of the generated dienes under various conditions. Conventional transition theory could not explain the corresponding reaction kinetics or stereochemical distributions and hence they employed ab initio steered molecular dynamics simulations. This revealed non‐equilibrium dynamic effects where the kinetic energy transduction generated from the first cycloelimination accelerated the following in one reaction step.^[^ ^64^ ^]^

Alongside, the force threshold of ladder‐type polymechanophores was studied in detail by single molecule force spectroscopy (SMFS) and computational simulations. This revealed the structure‐reactivity relationships of three reported ladder‐type cyclobutane mechanophores, including *exo*‐ladderane/ene, *endo*‐benzoladderene, and *exo*‐bicyclohexene‐*peri*‐naphthalene. It was found that threshold activation force and transition states are not sensitive with regard to the ring fused to the cyclobutane, but strongly depend on the steric and electronic properties of the first cleaved cyclobutane bond.^[^ ^65^ ^]^

A very recent enhancement of the polyladderene family was provided with the synthesis of fluorinated polyacetylene mechanophores by incorporating fluorine atoms into ladderene monomers by a cascade photochemical reaction. Under force activation, a gold‐colored, semiconducting fluoropolymer was produced.^[^ ^66^ ^]^

Polyladderene mechanophores present various unique and impactful features, in particular functional mechanochemical polymer‐to‐polymer transformation. Importantly, this technology provided an unprecedented method to generate *π*‐conjugated polyacetylene by force. The concluded fundamental investigations of ladder‐type mechanophores might inspire the design of other multicyclic multimechanophore structures.

## 5 Mechanosynthesis of Hexabenzocorones, Related Carbon Materials, and Allotropes

### 5.1 Hexabenzocorones and Scholl‐Type Reactions

**Scheme 4:**
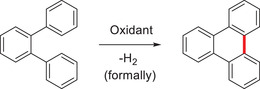
Simplified overview of a Scholl‐type reaction.

PAHs, nanographene, and graphene nanoribbons (GNRs) are of interest in material science by virtue of their unique properties.^[^ ^67^ ^]^ As defined magnetic and (opto)electronic properties rely on the width and configuration of corresponding materials,^[^ ^68^ ^]^ synthetic protocols need to fulfill the requirements of high accuracy and reproducibility.^[^ ^67b^ ^]^ These stipulations are matched by bottom‐up approaches, such as the Scholl reaction (**Scheme** **4**).

Discovered already around the 1910s,^[^ ^69^ ^]^ this class of reactions recaps the Lewis acid‐promoted coupling between arenes either intra‐ or intermolecularly. Since these pioneering reports, it found manifold applications in organic synthesis. For example, the role of FeCl_3_ in oxidative C—C coupling reactions has already been reviewed,^[^ ^70^ ^]^ and its fundamental role in the synthesis of hexabenzocoronenes or related polyarenes been discussed.^[^ ^71^ ^]^

Even though this oxidative dehydrogenative coupling reaction grants access to defined PAHs, one of its main constraints is the built‐in low solubility of the products. The surplus of arenes provokes distinct *π*–*π* interactions leading to agglomeration and low solubility,^[^ ^72^ ^]^ which is a pertinent problem for the synthesis and application of PAHs in materials science.

**Scheme 5:**
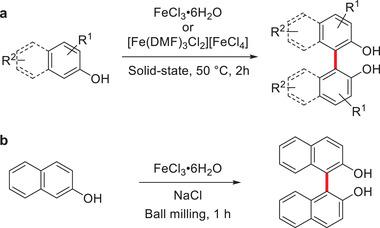
Oxidative coupling reactions of a) phenols in the solid‐state reported by Toda and coworkers^[^ ^73^ ^]^ and b) mechanochemical synthesis of BINOL.^[^ ^74a^ ^]^

In 1989, Toda and coworkers laid the groundwork for the mechanochemical synthesis of such structures when they reported the oxidative coupling reaction between phenols and Fe^III^ reagents in the solid‐state (**Scheme** **5**).^[^ ^73^ ^]^ During their investigation, the authors found that the reaction proceeds faster in the solid‐state compared to solution and could be further accelerated by the use of sonication. In addition, a cleaner reaction was achieved compared to many byproducts in solution. Motivated by these findings, Axelsson and coworkers reported the first mechanochemical synthesis of racemic BINOL (1,1′‐binaphthol).^[^ ^74^ ^]^ As the controlled heating of powders can be difficult, an alternative solid‐state synthesis was desired. 2‐Naphthol and FeCl_3_·6H_2_O were ground in the presence of NaCl for 1 h to obtain the desired product in 87% yield. The addition of NaCl was necessary to improve the rheology during the milling process. Using a ball mill, the reaction mixture was well mixed and slightly heated at the same time leading to a reproducible reaction. These first works laid the groundwork for the research of solvent‐free C—C bond formation reactions in ball mills,^[^ ^12^, ^14^, ^75^ ^]^ and further for the mechanochemical synthesis of carbon‐rich *π*‐conjugated materials discussed below.

#### 5.1.1 Hexabenzocoronene

**Scheme 6:**
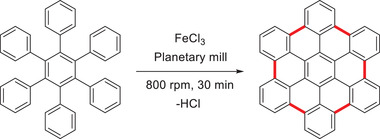
Mechanochemical Scholl reaction for the synthesis of HBC developed by Borchardt and coworkers.^[^ ^72^ ^]^

Knowing well how to handle insoluble products in the ball mill from a previous work about hyperbranched polyphenylenes,^[^ ^76^ ^]^ Borchardt and coworkers aimed for the development of a mechanochemical Scholl reaction.^[^ ^72^ ^]^ As a result, a solvent‐free protocol for the synthesis of the benchmark hexabenzocoronene (HBC) with FeCl_3_ as oxidant was developed (**Scheme** **6**).^[^ ^70^, ^71^ ^]^ Under optimized conditions, the authors were able to synthesize HBC in over 90% yield within a short milling time of 30 min. It was proven that chlorinated side‐products were suppressed by the correct choice of milling material as well as a reduced reaction time. The required quality and homogeneity of the products were demonstrated by UV–Vis spectroscopy and mass spectrometry. In the next step, the developed protocol was extended to larger PAHs, such as triangular‐shaped C_60_ and C_222_, where the authors obtained yields of 81% and 89%, respectively.^[^ ^72^ ^]^ As these are non‐optimized conditions, the results underline the advantages of mechanochemical Scholl reactions. They outperform classical solution approaches in yield (C_60_: 71%;^[^ ^77^ ^]^ C_222_: 62%^[^ ^78^ ^]^), reaction time (30 min versus 24 h),^[^ ^78^ ^]^ and allow the suppression of undesired side‐products.

**Figure 5:**
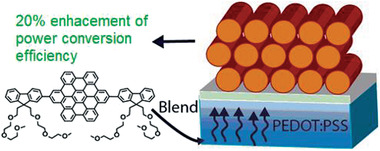
Templating strategy for performance enhancement of OPVs by Wong, Jones, and coworkers. This derivative was not synthesized mechanochemically but demonstrates the scope of application of this material class. Reproduced with permission.^[^ ^67f^ ^]^ Copyright 2014, American Chemical Society.

Despite these advantages, the developed procedure has two drawbacks. The use of corrosive solids (FeCl_3_) and the HCl generated during the reaction raise preparative problems and limit the scope. Therefore, the same group looked for a synthetic procedure to overcome these deficiencies. Motivated by the thermally activated cyclodehydrogenation conducted on a metal surface in electron microscopes,^[^ ^79^ ^]^ they examined the possibility of a Cu^0^‐promoted mechanochemical cyclodehydrogenation protocol.^[^ ^80^ ^]^ Studying the planarization of *o*‐terphenyl to triphenylene as a model system (compare Scheme 4), the authors had problems achieving a decent yield in a standard planetary mill (Fritsch Pulverisette 7 premium line planetary ball mill) operating at 800 rpm. A breakthrough was achieved, when the reaction was performed at 1500 rpm for 12 h in a high‐energy ball mill (Retsch Emax ball mill). As both mills have different geometries, a direct comparison of the conditions is difficult. However, the planetary mill uses mostly shearing forces, whereas the Emax combines impact and shearing forces due to its unique mode of action, allowing a higher energy input compared to standard mills. After further optimization and changing the milling material from zirconium oxide to dense tungsten carbide, a quantitative yield of triphenylene was obtained. Next, the developed mechanochemical protocol was applied in the synthesis of HBC, which was obtained in a quantitative yield, too.^[^ ^80^ ^]^ Even though a prolonged reaction time was needed compared to the mechanochemical Scholl protocol,^[^ ^72^ ^]^ in terms of used reagents, the procedure was milder giving rise to another mechanochemical graphitization tool omitting side‐reactions, for example, chlorination or dimerization, as well. The use of mechanosynthesis in the preparation of HBC and related materials can lead to technical progress in material science as HBCs (derivatives) have been successfully applied in supramolecular chemistry^[^ ^67c,d^ ^]^ organic electronics,^[^ ^67c,e^ ^]^ and organic photovoltaics (OPVs).^[^ ^67f,g^ ^]^ In the latter, a promising example was reported by Wong, Jones, and coworkers (**Figure** **5**).^[^ ^67f^ ^]^

As charge production and transportation are strongly influenced by the morphology of the active layer, they investigated the influence of a templating interlayer between the active layer and the electrode. The active layer in the tested OPV consisted of either 2,11‐bis(9,9‐dioctyl‐9*H*‐fluorene‐2‐yl)hexabenzo‐[bc,ef,hi,kl,no,qr]coronene (FHBC) or a mixture of FHBC and [6,6]‐phenyl C_61_‐butyric acid methyl ester (PCBM). Poly(3,4‐etyhlenedioxythiophene):poly(styrenesulfonate) (PEDOT:PSS), a conducting polymer, was selected as electrode. Applying the templating strategy, the authors wanted to change the domain sizes, crystal packing, or the orientation of molecules, hoping to improve the device's performance. To achieve this goal different HBC derivatives have been applied as interface modifiers, which were blended into the PEDOT:PSS‐electrode. Not only did a small amount of interface modifiers (0.2 wt%) during the blending lead to a change in the morphology, but also increased the photovoltaic performance by over 20%. As HBCs are being explored in the field of supramolecular electronics and show promising electrical properties,^[^ ^67c^ ^]^ the efficient, effortless scalable synthesis of PAHs by mechanochemistry can be key for future developments as the one described above.

#### 5.1.2 Scholl Polymerization

**Scheme 7:**
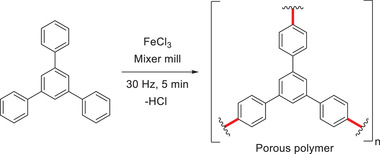
FeCl_3_‐mediated mechanochemical Scholl polymerization reported by Borchardt and coworkers.^[^ ^81^ ^]^

Continuing the research on the FeCl_3_‐mediated mechanochemical Scholl reactions, the group of Borchardt aimed to broaden its application towards a mechanochemical Scholl polymerization reaction (**Scheme** **7**).^[^ ^81^ ^]^ After a short optimization, the desired porous polymer was obtained in an almost quantitative yield using a mixer mill for only 5 min to react the monomer in the presence of FeCl_3_. The high purity of the product was ensured by elemental analysis and Rutherford backscattering spectroscopy. Further analysis of the polymer showed a high specific surface area of 658 m^2^ g^−1^. Employing LAG, it was increased to 1090 m^2^ g^−1^, which was comparable to a sample prepared in solution. In their findings, the authors proved that the porosity of the prepared polymer and the pore geometry was strongly dependent on the mechanochemical process itself, for example, milling time, frequency, milling material, LAG, and the used monomers. In addition, the use of a ball mill simplified the Scholl polymerization as inert atmosphere, solvents, or multistep syntheses were avoided.^[^ ^81^ ^]^

### 5.2 Porous Networks and Porous Carbon

Since the synthesis of microporous poly(aryleneethynylene) networks was established by Cooper and coworkers,^[^ ^82^ ^]^ conjugated porous networks (CPNs) are being studied extensively.^[^ ^83^ ^]^ Comparable to the polymeric materials obtained by the Scholl reaction, CPNs exhibit nanoporous skeletons, large specific surface areas, and diverse structures rendering them valuable alternatives to metal‐organic frameworks (MOFs) as candidates for gas separation, (energy) storage, sorbent materials, versatile catalysts, or molecular sensors.^[^ ^82^, ^83^ ^]^ Since an overview of the mechanosynthesis of porous organic materials is available,^[^ ^84^ ^]^ only publications from 2017 and onwards will be discussed here.

Besides the already discussed example of a mechanochemical oxidative Scholl polymerization (Scheme 7), another example for a mechanosynthesis of a CPN was reported in 2017 by Borchardt and coworkers. They applied AlCl_3_‐mediated Friedel–Crafts alkylations between cyanuric chloride and electron‐rich aromatic compounds.^[^ ^85^ ^]^ For instance, the combination of carbazole and cyanuric chloride led to a quantitative yield after 1 h of milling giving access to a CPN with a specific surface area of 570 m^2^ g^−1^ and defined pore size distribution ranging from 0.5–1.0 nm. Using this protocol, even unreactive monomers, such as benzene, reacted to some extent and the authors demonstrated the scalability of the procedure (reactions were performed with a total mass loading of 10 g) granting access to a wide variety of CPNs.

**Scheme 8:**
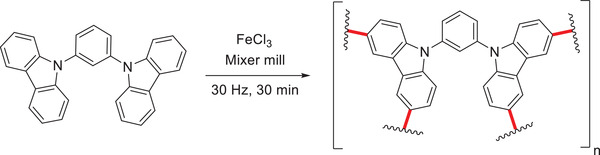
Mechanosynthesis of a CPN based on a carbazole backbone by Dai and coworkers.^[^ ^86^ ^]^

Having proven the synthesis of porous networks under mechanochemical conditions, another example was reported by Dai and coworkers in the same year.^[^ ^86^ ^]^ This protocol was also based on a carbazole scaffold but used an oxidative coupling strategy instead. The authors milled 1,3‐bis(*N*‐carbazolyl)benzene in the presence of FeCl_3_ in a mixer mill for 30 min at a frequency of 30 Hz (**Scheme** **8**) to obtain the desired CPN in 83% yield. Brunauer–Emmett–Teller (BET) analysis revealed a specific surface area of 707 m^2^ g^−1^ outperforming solution protocols (483 m^2^ g^−1^).^[^ ^87^ ^]^ Even though both CPNs by Borchardt and Dai are based on a carbazole backbone, it was reasoned that the difference in the BET surface and porosity stemmed from a different linking efficiency between the Friedel–Crafts and the oxidative coupling approach. After the developed protocol was successfully applied to other substrates, the CO_2_ storage abilities were investigated. With a CO_2_ storage capacity of 92.3 cm^3^ g^−1^, many nanoporous polymers described in the literature are surpassed.^[^ ^86^ ^]^ The authors could show that the yield and porosity were closely related to the amount of FeCl_3_ used as well as the milling time. Thereby, CPNs with tailor‐made porosity can be accessed simply by varying the milling time or changing the amount of mediator.^[^ ^86^ ^]^

**Scheme 9:**
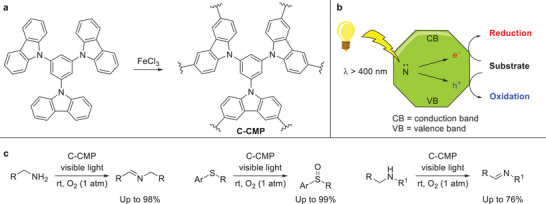
C‐CMP catalyzed photoredox oxidations reported by Loh and coworkers.^[^ ^83g^ ^]^ a) Preparation of the C‐CMP. b) Schematic representation of C‐CMP as photocatalyst. c) Visible light photocatalysis of aerobic oxidation reactions using C‐CMP.

The value and versatility of carbazolic conjugated microporous polymers (C‐CMP) can be inferred from their photocatalytic properties.^[^ ^83f^ ^]^ Loh and coworkers reported the visible light photocatalysis of aerobic oxidation reactions using C‐CMP (**Scheme** **9**).^[^ ^83g^ ^]^ The polymer was prepared by polymerizing 1,3,5‐tri(9‐carbazolyl)benzene using a Scholl‐type reaction. After purification, the C‐CMP was evaluated as photocatalyst for several oxidation reactions. Primary amines were oxidized to their corresponding imines in excellent yields and sulfides to their sulfoxide derivatives with high chemoselectivity. Even though this result shows the significance of such polymers in material science, its preparation is solution‐based and requires a tedious workup and substantial amounts of solvent. As this system is closely related to the ones described by Borchardt and Dai,^[^ ^85^, ^86^ ^]^ mechanochemistry could be applied to make the syntheses of C‐CMPs even more attractive.

Dai and coworkers continued their work on the mechanochemical synthesis of nanoporous polymer networks (NPNs), especially the FeCl_3_‐mediated oxidative coupling polymerization. In 2018, they reported the membrane‐accelerated CO_2_ separation using 3,3,3′,3’‐tetramethyl‐2,2′,3,3′‐tetrahydro‐1,1′‐pirobi[indene]‐6,6′‐diol as monomer for the nanoporous polymer network (NPN) and 2,2‐bis(3,4‐dicarboxyphenyl)‐1,1,1,3,3,3‐hexafluoropropane dianhydride/diaminomesitylene as the polymer matrix.^[^ ^88^ ^]^ The application of mechanochemistry was key for the successful separation of a CO_2_/CH_4_ mixture. When the porous polymer was prepared by a solution protocol it was insoluble in common organic solvents, while its mechanochemically prepared counterpart dissolved and was of high quality. Thus, the authors reasoned that the mechanochemically oxidative polymerization is subject to an inherent molecular weight control over polymer growth, resulting in a high solubility of the material. Again, the authors proved that yield and porosity correlated to milling time and amount of FeCl_3_. Moreover, the sample dispersed homogeneously within a membrane leading to high CO_2_ permeability and a CO_2_/CH_4_ selectivity of 25.^[^ ^88^ ^]^

In 2020, Dai and coworkers reported a storage material for (radioactive) organic iodide prepared by mechanosynthesis.^[^ ^89^ ^]^ The microporous organic polymer was obtained by grinding triptycenehexamine together with pillar[5]quinone for 30 min at 30 Hz in high yield, regardless of the poor solubility of the quinone, showing once more that mechanochemistry can circumvent solubility issues.

The porous materials described so far have been used mostly for (gas) storage,^[^ ^86^ ^]^ separation,^[^ ^88^ ^]^ and catalysis.^[^ ^83^, ^87^ ^]^ Recently, Dai and coworkers reported a mechanochemical protocol that does not rely on the use of noble metal complexes, Lewis acids, electron‐rich substrates, or ultrahigh‐vacuum environment at high temperatures for the synthesis of CPNs.^[^ ^90^ ^]^ Instead, halogenated arenes and Mg are used to perform reductive dehalogenation by an Ullmann‐type coupling. After the arenes are knitted together and depending on the used monomer, porous materials consisting of meso‐ and macropores or highly crystalline graphite structures were obtained. For the mechanochemical reaction, the authors presumed a reaction pathway similar to the one described for a surface synthesis. They reasoned that the provided mechanical energy would be sufficient to start the insertion of Mg species into the C—X bond, resulting in a rapid and efficient solid‐state formation of the final CPN by a reductive Ullmann‐type coupling. However, being a mild and straightforward synthesis compared to other methods described,^[^ ^90^ ^]^ the influence of mechanochemistry on the material properties was only discussed briefly. For instance, a small particle size, conjugated architecture, permanent porosity, and a robust network were mentioned, but only the degree of polymerization as well the porosity were correlated directly with the milling time. Nonetheless, due to their unique features, the as‐prepared CPNs have been evaluated as electrode materials in Li‐ion batteries and showed long‐term cycling stability and a rate performance superior to natural graphite making them promising candidates as electrode materials.^[^ ^90^ ^]^

Another class of porous materials that has been used for electrochemical energy storage, is (doped) nanoporous carbon.^[^ ^91^ ^]^ In this context, Zhang, Wang, and coworkers described a ball mill‐assisted synthesis thereof.^[^ ^92^ ^]^ The preparative basis is a mechanochemical mixing of urotropine and FeCl_3_·6H_2_O prior to carbonization at 850 ℃. The mechanochemical treatment of hexamethylenetetramine with different equivalents of FeCl_3_ was used to prepare different precursors for porous carbon materials leading to different morphologies and graphitization degrees after carbonization. The obtained porous carbon samples differed in terms of specific surface area, pore volume, and pore width resulting in distinct electrochemical properties. However, the influence of mechanochemistry on the system was not discussed in detail and remained unclear.^[^ ^92^ ^]^

**Scheme 10:**
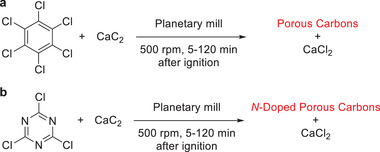
Mechanochemical synthesis of a) porous carbon and b) *N*‐doped porous carbons by Borchardt and coworkers.^[^ ^93^, ^94^ ^]^

Borchardt and coworkers reported a mechanochemical synthesis of porous carbon with a specific surface area up to 915 m^2^ g^−1^.^[^ ^93^ ^]^ The procedure is based on a chemical reaction between calcium carbide and hexachlorobenzene (**Scheme** **10**). After a short mechanical activation, the coupling of the two reagents occurs as combustion due to the highly exothermic process. Different ratios of chemicals as well as alterations in milling time yielded carbon materials with different specific surface areas and porosities. With increasing milling time and a CaC_2_/C_6_Cl_6_ ratio, porosity and specific surface were increased. At a low stoichiometric ratio of 0.9, mostly graphene stacks were formed, exhibiting almost no porosity, whereas a ratio of 5.1 gave rise to the already mentioned high specific surface area, due to the presence of spherical‐shaped carbons and nanosized strips.^[^ ^93^ ^]^ The published protocol does not only offer the advantage of a solvent‐free synthesis but also takes place in the absence of heating or activating agents. Moreover, it uses a chlorinated pollutant as a reagent, which is converted to environmentally benign CaCl_2_ and a valuable carbonaceous material potentially allowing upcycling by mechanochemistry.

**Figure 6:**
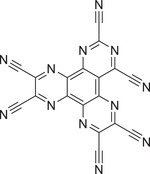
Structure of the multipurpose molecule HAT‐CN.^[^ ^99^ ^]^

Considering the advantages of *N*‐doped porous carbon materials, for example, improved reversible capacity, rate capability, long‐term cycling stability, and wettability,^[^ ^95^ ^]^ Borchardt and coworkers explored the mechanosynthesis of such materials. In a first study,^[^ ^96^ ^]^ lignin was used as carbon source and urea served as nitrogen doping reagent. After milling of the chemicals, the resulting polymer was carbonized. In this case, milling was mandatory for two reasons. First, the nitrogen incorporation was exclusively recorded under mechanochemical conditions. Thereby, a lignin polymer containing pyrrolic/pyridonic and quaternary nitrogen groups was formed. Second, potassium carbonate was used to induce a carbothermal reduction during the carbonization process leading to the introduction of pores as carbon atoms were removed.^[^ ^97^ ^]^ Thus, ball milling promoted a homogenous distribution of K_2_CO_3_. Following the published protocol, the authors could not only obtain an *N*‐doped porous carbon material with a specific surface area of 3041 m^2^ g^−1^ and a nitrogen content of 6.3 wt%, but also show an outstanding performance of the material as an electrode. However, as this procedure required carbonization at high temperatures, the group explored the possibility of a mechanically induced self‐sustaining reaction (MSR) for *N*‐doped porous carbon (Scheme 10).^[^ ^94^ ^]^ Descending from self‐propagating combustion reactions, where a reaction is initiated for instance by a spark or heat coil, and continues spontaneously due to its highly exothermic nature, an MSR is set to get the ball rolling using mechanical activation.^[^ ^98^ ^]^ They found such a system in the combination of cyanuric chloride and CaC_2_. The authors analyzed the ratio between triazene and calcium carbide and found that the presence of cyanuric chloride was crucial for the ignition as no reaction took place when pure carbide was ball milled. Moreover, the yield increased with higher CaC_2_ content but was accompanied by a decrease in nitrogen content. It was found that an excess of carbide was beneficial as it promoted the reaction rapidly and most of the product was formed instantly during the ignition (1–5 min reaction time). Furthermore, a prolonged milling time was helpful, since it did not only enable a continuous particle size reduction leading to a better purification but also the mechanical milling was essential for the outcome of the reaction as it was used for homogenization and increase of structural defects as well as particle surface. Recently, they reported the mechanochemically assisted synthesis of 1,4,5,8,9,11‐hexaazatriphenylenehexacarbonitrile (HAT‐CN),^[^ ^99^ ^]^ a highly *N*‐doped carbonaceous material with a C:N ratio of 3:2 bearing no hydrogen atoms (**Figure** **6**). Offering a multipurpose use, for example, in the preparation of highly *N*‐doped carbons,^[^ ^100^ ^]^ or organic light‐emitting diode (OLED) architectures,^[^ ^101^ ^]^ it is an encouraging molecule for material sciences. Performing LAG between hexaketocyclohexane and diaminomaleonitrile using H_2_O for 10 min in a mixer mill and subsequent treatment of the condensation product with HNO_3_ gave the product in 67% yield. Thus, the mechanochemically assisted protocol outperforms the state of the art with respect to both the yield and the green metrics as the heating treatments, solvent usage, and over‐stoichiometric use of chemicals were reduced.^[^ ^99^ ^]^

The selected examples show how mechanochemistry can be used for the synthesis of porous materials. Its implementation leads to high yields of high‐quality products with tailor‐made properties and enables the achievement of results that would be otherwise impossible. Especially in the handling of insoluble materials ball milling demonstrates its strength.

### 5.3 Graphene‐Related and Other Carbon Allotropes

Since its groundbreaking discovery in 2004 by Novoselov and Geim,^[^ ^102^ ^]^ graphene has been studied elaborately, due to its unique properties and applications in electronics.^[^ ^67^, ^102^, ^103^ ^]^ The honeycomb structure of graphene consisting of sp^2^‐carbons only can be considered as a precursor for other carbon allotropes. For instance, when the 2D sheet is wrapped, it can be deemed as 0D fullerene or stacked as 3D graphite.^[^ ^103b^ ^]^ Therefore, the mechanochemical synthesis of fullerenes, graphynes, and selected other carbon allotropes/carbon‐rich materials will be reviewed. However, the mechanochemistry of graphene itself will not be discussed as reviews already exist in the literature.^[^ ^103e–m^ ^]^

#### 5.3.1 Fullerenes

**Scheme 11:**
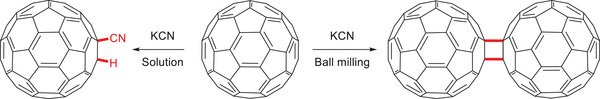
Different product selectivity achieved in solution and by mechanochemistry.^[^ ^70^, ^104^, ^105^ ^]^

In 1997, Wang, Komatsu, and coworkers reported a seminal mechanochemical synthesis of the fullerene dimer C_120_.^[^ ^3^, ^104^ ^]^ Milling of C_60_ in the presence of additives, for example, KCN, alkali metals, or 4‐aminopyridine, for 30 min at 3500 rpm in an inert atmosphere led to C_120_ in 30% yield. The reaction was thoroughly studied by the authors. Besides the discovery of an equilibrium between C_60_ and C_120_ with a ratio of 7:3 in weight, two mechanisms for the formation of the “bucky dumbbells” were discussed. Both mechanisms involve a prior attack by the cyanide anion. Subsequently, the dimerization proceeds either by a nucleophilic addition pathway or a coupling by an electron transfer reaction leading to a chain reaction. The later reaction pathway is discussed to be more probable as lesser amounts of alkali metal allow the dimerization. This study is one of the most prominent examples of how mechanochemistry can alter product selectivity,^[^ ^70a^ ^]^ as the solution protocol would lead to functionalization of C_60_ and not the dimerization (**Scheme** **11**).^[^ ^105^ ^]^

**Figure 7:**
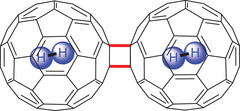
Endohedral fullerene dimer (H_2_@C_60_)_2_.^[^ ^104^ ^]^

In 2005, Komatsu and coworkers used the described mechanosynthesis for the preparation of endohedral fullerene C_120_ containing molecular hydrogen (**Figure** **7**).^[^ ^104^ ^]^ These extraordinary molecules are of interest as they do not only act as containers for (reactive) molecules free from (significant) interactions with the surrounding medium but also allow their extensive study due to their stability.^[^ ^106^ ^]^ The authors developed a four‐step synthesis to completely close a 13‐membered ring orifice. The chosen method enabled the encapsulation of H_2_ and resulted in a satisfactory yield compared to other methods which relied on physical methods, such as co‐vaporization, high pressure, and temperature treatment requiring tedious isolation. Using the developed method of ring closure, H_2_@C_60_ was obtained in 60% yield. To study its reactivity, the mechanosynthesis of C_120_ described before was applied,^[^ ^3^ ^]^ and (H_2_@C_60_)_2_ was prepared in 30% yield. NMR analysis revealed that the reactivity of H_2_@C_60_ was not influenced by the encapsulated hydrogen.

**Scheme 12:**
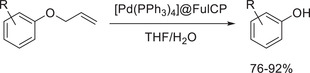
Deallylation of allyl phenyl ethers by a Pd catalyst impregnated on a fullerene‐bearing porous polymer prepared by ball milling.^[^ ^108^ ^]^

After the prominent example of Komatsu and coworkers, mechanochemistry of fullerenes and related materials has become a growing field, being reviewed in the literature, recently.^[^ ^107^ ^]^ A notable example is the work of Chen, Feng, Sui, and coworkers combining fullerenes and porous polymers.^[^ ^108^ ^]^ A porous polycarbazole polymer containing fullerene (FulCP) was investigated as a carrier material for a heterogeneous catalyst. The porous polymer was prepared by both solution and mechanochemical approaches connecting fullerene‐carbazole precursors in the presence of FeCl_3_. On one hand, the mechanochemically prepared polymer offered a larger specific surface area than the one prepared in solution (1015 versus 920 m^2^ g^−1^), on the other hand, the mechanochemical protocol did not result in the chlorination of the phenyl rings, a side reaction that was observed for the solution counterpart. However, the authors did not provide a reason why the mechanochemical reaction was cleaner. It could only be hypothesized that the use of chloroform as solvent and concentrated hydrochloric acid during the work‐up procedure may explain the side reactions for the solution protocol. Having identified the ball milling approach as suitable for the synthesis of the porous polymer, it was impregnated with (Pd(PPh_3_)_4_). The obtained heterogeneous catalyst was then applied in the deallylation of allyl phenyl ethers (**Scheme** **12**). It showed good recyclability, and the corresponding phenols were obtained in good to excellent yields. The performance is comparable to other known catalysts. However, the synthesis of such catalysts is laborious and not easily carried out on larger scales.

#### 5.3.2 Graphynes

**Figure 8:**
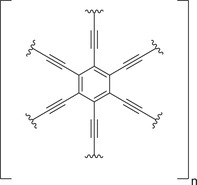
Repeating unit of *γ*‐graphyne.

Graphene, being the most prominent and widely studied member of 2D carbon allotropes/materials,^[^ ^103e–m^ ^]^ found multiple applications in electrochemistry as anode material, especially in Li‐ion batteries. Advantages, such as light weight, stability, and conductivity, are highly appreciated.^[^ ^109^ ^]^ However, consisting exclusively of sp^2^‐carbons, the material can store Li^+^ only between stacked layers/sheets leading to a limited Li^+^ storage mechanism.^[^ ^110^ ^]^ To overcome this issue, graphynes, particularly *γ*‐graphynes (**Figure** **8**), are discussed as a next‐generation Li‐ion anode material,^[^ ^111^ ^]^ as they combine sp‐ and sp^2^‐carbons offering access to large conjugated rings together with being planar resulting in a higher capacity compared to graphite materials.

Already in 1987, graphynes have been proposed theoretically by Baughman, Eckhardt, and Kertesz,^[^ ^112^ ^]^ but the first mechanosynthesis dates to 2018. The group of Cui investigated the synthesis of *γ*‐graphyne and its electronic structure.^[^ ^113^ ^]^ To obtain the desired carbonaceous material, the authors milled hexabromobenzene and calcium carbide. An approach comparable to the one by Borchardt and coworkers (Scheme 10).^[^ ^93^, ^94^ ^]^ However, they added EtOH to their milling vessel and milled the reaction mixture for 16 h at 450 rpm, with cooling breaks every 15 min to avoid overheating. Therefore, it is likely that they could prevent the ignition event reported by Borchardt and did not aim for an MSR.^[^ ^94^ ^]^ Following this protocol, a conversion of 80% of hexabromobenzene was observed and the prepared graphyne was analyzed by several techniques. Besides being a monocrystalline nanosheet with a lattice constant of 0.69 nm, it showed high thermal stability, which could even be considered as partially incombustible. Moreover, a potential application in photocatalytic degradation or oxygen evolution reactions (OER) was discussed as it offered a high absorbance in the UV–Vis region.^[^ ^113^ ^]^ In a follow‐up study, the authors altered the mechanochemical preparation slightly to obtain a mesoporous *γ*‐graphyne. EtOH was removed from the milling process, and the resulting reaction mixture was calcinated for 2 h at 450 ℃ to remove structural defects. This material provided distinguished electrochemical properties resulting from its high structural integrity, large conjugated rings, and mesoporous design.^[^ ^114^ ^]^ In the same year, the group continued their work using *γ*‐graphyne as an electrocatalyst for oxygen evolution reactions (OER).^[^ ^115^ ^]^ Utilizing benzene and calcium carbide as precursors and following their first reported protocol,^[^ ^113^ ^]^ they developed a scalable synthesis of this potential electrocatalyst. In the area of renewable energies and H_2_O‐splitting being a key reaction, these findings can pave the way for extensive application of *γ*‐graphyne in manifold areas.^[^ ^115^ ^]^

**Scheme 13:**
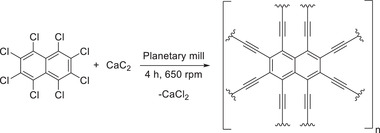
Mechanochemical synthesis of naphyne by Li, Liu, Li, and coworkers.^[^ ^116^ ^]^

Concerning the successful application of calcium carbide and perhalogenated benzene derivatives or benzene in the mechanosynthesis of graphyne,^[^ ^113^, ^114^, ^115^ ^]^ the introduction of naphthyl units was foregone. In 2020 Li, Liu, Li, and coworkers reported the mechanochemical synthesis of an alkynyl‐linked naphthyl carbon skeleton: naphyne.^[^ ^116^ ^]^ The authors reacted perchloronaphthalene and calcium carbide in a planetary mill for 4 h at 650 rpm under vacuum with implemented cooling intervals of 5 min every 30 min to yield the desired material in 98% yield (**Scheme** **13**).

In this study, the authors discussed the impact of mechanical energy on the reaction. They concluded that milling breaks up the crystalline structure of CaC_2_ releasing nucleophilic C_2_^2−^. After the first substitution on the naphthyl derivate, the reaction proceeds rapidly due to the electron‐withdrawing abilities of the alkynyl groups leading to complete dechlorination and CaCl_2_ formation. Extensive solid‐state analysis revealed the morphology of the prepared polycrystalline naphyne and was described as cross‐linked sheets with sp^2^‐ and sp‐carbons being the dominating hybridization types. The electrochemical properties of naphyne were excellent as the capacity retention was 92% after 5000 charge–discharge cycles.^[^ ^116^ ^]^

#### 5.3.3 Chains and Curves

**Scheme 14:**
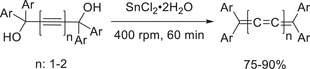
Mechanosynthesis of [n]cumulenes.^[^ ^117^ ^]^

Besides its successful application in the synthesis of aromatic or planar carbon allotropes, mechanochemistry was successfully applied in the synthesis of linear or curved molecules, such as carbyne‐type precursors or curved aromatics. In 2019, Hernández and coworkers reported the mechanochemical synthesis of odd‐numbered tetraaryl[n]cumulenes.^[^ ^117^ ^]^ This compound class is of importance as cumulenes are ideal model compounds to study the properties of carbyne,^[^ ^118^ ^]^ a carbon chain consisting only of sp‐carbons of infinite length and theoretically discussed to be the strongest known material. At the outset, the authors investigated a mechanochemical Favorskii alkynylation‐type reaction between ketones and calcium carbide as alkyne source. In this manner, propargylic diols were formed as major products, which were then used in a mechanochemical, solvent‐ and acid‐free reductive elimination with SnCl_2_ (**Scheme** **14**). Ball milling the corresponding diols in the presence of SnCl_2_ gave access to [3]‐ and [5]cumulenes in good to excellent yields. Even though the physicochemical properties of the products have not been analyzed, the study provides the basis for further investigations of reliable and scalable ways of carbyne formations. Even more, as the protocol is solvent‐ and acid‐free, poorly soluble or acid‐sensitive substrates can be applied.

**Scheme 15:**
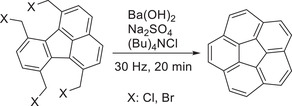
Mechanochemical synthesis of corannulene by García, Stuparu, and coworkers.^[^ ^121^ ^]^

It is strikingly shown by fullerenes or nanotubes that carbon‐rich materials are not inevitably flat. Besides doping or (edge) functionalization, curvature can be a means to an end to induce defects in materials or even to introduce chirality,^[^ ^119^ ^]^ aiming for different properties.^[^ ^119^, ^120^ ^]^ Synthesizing these strained molecules usually requires long reaction times, results in low yields, or has a high power demand requiring flash vacuum pyrolysis (FVP) to proceed.^[^ ^120^, ^121^ ^]^ With respect to FVP, the precursors must fulfill the requirement of being able to sublimate and withstand harsh conditions. However, mechanochemical syntheses are not limited by such demands. In contrast, scale‐up, shorter reaction times, and a straightforward setup were used by García, Stuparu, and coworkers for the mechanochemical synthesis of corannulene.^[^ ^121^ ^]^ The authors sought an alternative synthetic route based on impact and shear forces to populate the bent geometries needed for the preparation of curved molecules. The study focused on tetrabromomethylfluoranthene as a precursor allowing the direct comparison between FVP, solution, and mechanochemical syntheses of corannulene.^[^ ^121^, ^122^ ^]^ The curved molecule was obtained in a short reaction time of 20 min at a frequency of 30 Hz by milling Ba(OH)_2_, tetrabutylammonium chloride, Na_2_SO_4_, and the aromatic precursor (**Scheme** **15**). The pure product was obtained in 60–67% yield, which outperforms the yields achieved by FVP (18%)^[^ ^122a^ ^]^ and in solution (14%)^[^ ^122b^ ^]^ significantly. Moreover, the authors were able to gain inside the mechanism and applied mechanosynthesis in the preparation of tetrabromofluoranthene derivates. Thus, the overall synthetic protocol became more environmentally benign as reaction times, stoichiometric amounts, and the need for solvents were optimized.

**Figure 9:**
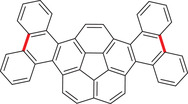
Backbone of the corannulene‐phenanthrene hybrid material.^[^ ^123^ ^]^

In another study, García, Stuparu, and coworkers reported the mechanochemical functionalization of curved aromatics to yield corannulene‐based nanographenes.^[^ ^123^ ^]^ It was hypothesized that the synthesis of such a hybrid structure (**Figure** **9**) could not be prepared by photochemical annulation or oxidative coupling reactions in solution as the curvature of the structure would hinder such conversion.^[^ ^122b,c^ ^]^ Therefore, the hybrid material was only known theoretically. The key step towards the preparation of the corannulene‐phenanthrene hybrid was an FeCl_3_‐mediated mechanochemical Scholl reaction. After a short optimization that included the influence of temperature and the purity of starting material, the authors were able to obtain the hybrid in 97% after 15 min, and they could also demonstrate the applicability of the protocol on a large scale. This scale‐up was a key for the further study of the prepared material. The electrochemical properties turned out to be remarkable offering reversibility of the electron‐accepting properties as determined by cyclovoltammetry. Additionally, the prepared reduced radical anion was stable for days in solution.

**Figure 10:**
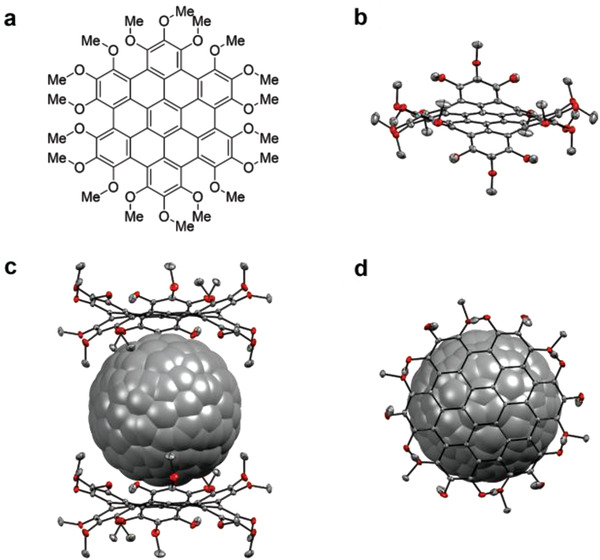
Curved HBC in supramolecular chemistry.^[^ ^71b^ ^]^ a) Structure of permethoxylated HBC. b) X‐ray crystal structure of permethoxylated HBC (CCDC‐245390). c) X‐ray crystal structure of host‐guest complex between permethoxylated HBC and C60 (CCDC‐245392) along the *b*‐axis and d) along the *c*‐axis. X‐ray crystal structures were obtained from the Cambridge Structural Database and visualized using Mercury.^[^ ^124^ ^]^

Such curved molecules are of high interest in supramolecular chemistry as they represent bowls, a highly desired structural motif in host‐guest complexes. Müllen and coworkers prepared a curved HBC using a FeCl_3_‐mediated Scholl reaction.^[^ ^71b^ ^]^ Due to the presence of 18 free‐rotating methoxy groups, the resulting HBC showed a double‐concave conformation (**Figure** **10**). It was used as a host molecule in a co‐crystallization approach together with hexafluorobenzene or C_60_ as guest molecules. The remarkable X‐ray crystal structure of the host‐guest complex with fullerene is illustrated in Figure 10c,d. The authors suggest a future application of these host‐guest molecules in the field of liquid crystals if the PAH or fullerene would be attached with long alkyl chains, for instance. As the mechanosynthesis of PAHs, fullerenes, and curved molecules was already described, and solution‐based protocols can be puzzling, mechanochemistry could be a potential game‐changer in this area.

## 6 Conclusion

Though being a fundamentally old technique, recent advancements in mechanochemistry fuel the continuous rapid development and growth of this field. Its use to synthetically prepare and activate *π*‐conjugated carbon‐rich materials offers an insight into strategies to mechanically manipulate chemical bonds to induce chemical reaction which cannot proceed using conventional physical stimuli, such as light or heat. We have provided ample highlight examples that prove the effects of force on the chemistry of carbon‐rich materials. Functional responses include the polymer mechanochemical activation of *π*‐systems for OFPs, the release of *π*‐conjugated dye molecules, the shear‐induced formation of polyacetylenes, and the bottom‐up facilitated and green syntheses of carbon allotropes by milling. Different magnitudes of forces are exerted on multiple scales ranging from single polymer chains in single molecule force spectroscopy (SMFS) over solution and powder processes to bulk polymers. Mechanochemistry proves its strength on all scales and on the one hand offers access to unprecedented functionalities in polymers using force as unique and orthogonal stimulus. On the other hand, synthetic procedures in mills overcome obstacles, such as substrate insolubility, solvent waste, long reaction times, low yields, and side‐product generation. Prospectively, even the upcycling of pollutants might be possible. Future works might be dedicated to the identification of unifying concepts in mechanochemistry amalgamating the rich functionalities achievable by polymer mechanochemistry with the synthetic bond formation prowess of trituration mechanochemistry. Thereby, we anticipate exciting new materials and their applications.

## Conflict of Interest

The authors declare no conflict of interest.
